# IL-37 Expression in Patients with Abdominal Aortic Aneurysm and Its Role in the Necroptosis of Vascular Smooth Muscle Cells

**DOI:** 10.1155/2022/1806513

**Published:** 2022-05-11

**Authors:** Yan Ding, Yue Wang, Yifan Cai, Chengliang Pan, Chao Yang, Miao Wang, Xiaoyu Qi, Jing Ye, Qingwei Ji, Jian Yu, Wenbin Xu, Kunwu Yu, Qiutang Zeng

**Affiliations:** ^1^Department of Cardiology, Union Hospital, Tongji Medical College, Huazhong University of Science and Technology, Wuhan, Hubei 430022, China; ^2^Department of Vascular Surgery, Union Hospital, Tongji Medical College, Huazhong University of Science and Technology, Wuhan, Hubei 430022, China; ^3^Department of Urology, Union Hospital, Tongji Medical College, Huazhong University of Science and Technology, Wuhan, Hubei 430022, China; ^4^Department of Cardiology, Renmin Hospital of Wuhan University, Wuhan, Hubei 430022, China; ^5^Department of Cardiology, The People's Hospital of Guangxi Zhuang Autonomous Region, Nanning 530021, China

## Abstract

**Background:**

Our previous studies have shown that interleukin- (IL-) 37 plays a protective role in patients and animal models with coronary artery disease. However, the role of IL-37 in patients with abdominal aortic aneurysm (AAA), another artery disease, is yet to be elucidated.

**Methods and Results:**

AAA tissues and plasma samples were obtained from patients with or without surgical intervention. Normal renal aortic tissues were collected from kidney transplant donors. Our findings established that in AAA, IL-37 was distributed in endothelial cells, macrophages, and vascular smooth muscle cells (VSMCs) and that it was chiefly concentrated in VSMCs. Furthermore, the expression was found to be downregulated compared with that in normal artery tissues. Immunofluorescence showed that, unlike normal arteries, IL-37 was translocated to the nucleus of VSMCs in AAA. Moreover, in patients with AAA, the expressions of IL-37, IL-6, and tumor necrosis factor- (TNF-) *α* were increased in the plasma in comparison with the healthy controls. Correlation analysis revealed that IL-37 was positively correlated with IL-6, TNF-*α*, age, aneurysm diameter, and blood pressure. Furthermore, human aortic vascular smooth muscle cells (HASMCs) were stimulated with angiotensin II (AngII) *in vitro* to simulate smooth muscle cell (SMC) damage in AAA. A decrease in IL-37 expression and an increase in receptor-interacting serine/threonine-protein kinase 3 (RIPK3) expression were observed in HASMCs stimulated with AngII. On this basis, inhibition of RIPK3 with GSK'872 significantly attenuated necroptosis. Moreover, the necroptosis rates were significantly lowered in HASMCs treated with recombinant IL-37, whereas the rates were enhanced when the cells were depleted of the interleukin. Immunoblotting results showed that both exogenous and endogenous IL-37 could affect the expressions of RIPK3, NLRP3, and IL-1*β*. Also, the phosphorylation of RIPK3 and p65 was affected. Meanwhile, IL-37 promoted the transition of SMC from proliferative type to contractile type.

**Conclusions:**

The expression of IL-37 in VSMCs decreases in patients with AAA, whereas IL-37 supplementation suppresses RIPK3-mediated necroptosis and promotes the transition of VSMCs from proliferative to contractile type.

## 1. Introduction

Abdominal aortic aneurysm (AAA) is a permanent and irreversible local expansion of the abdominal aorta, which exceeds 50% of the normal vessel diameter or is >3 cm. The majority of AAAs involve the abdominal aorta adjacent to the renal artery [1]. AAA is conventionally considered to be a localized phenotype of advanced atherosclerosis. However, presently, evidence that the entire blood vessel is abnormal in patients with AAA is mounting [2, 3]. Recent studies have uncovered that the pathophysiological process of AAA includes oxidative stress, apoptosis, inflammation, abnormal proteolytic pathways, and loss of arterial wall matrix [4]. No specific drug is currently available to treat AAA; surgery is the only effective option [5] and is resorted to in the terminal stages of the illness. Therefore, it is imperative to identify new drugs for the treatment of AAA, especially to help patients in the early stages of the disease. Interleukin- (IL-) 37, whose precursor is IL1F7, is a novel anti-inflammatory cytokine that belongs to the IL-1 ligand family [6]. As a class of molecules unique to humans, IL-37 is produced via proinflammatory stimulation to prevent inflammation resulting from overstimulation and protects the tissues from excessive damage [7]. Our earlier study has established that IL-37 is significantly increased in the serum and vascular smooth muscle cells (VSMCs) of patients with coronary atherosclerosis and arterial calcification [8]. Recombinant IL- (rIL-) 37 has been shown to alleviate mouse atherosclerosis and myocardial infarction by inhibiting cardiomyocyte apoptosis, a finding that asserts that IL-37 is inextricably linked to cardiovascular disease [9–11]. However, the association of IL-37 with AAA remains unknown.

Necrosis and apoptosis have previously been described as the two different forms of cell death [12, 13]. Apoptosis, which is programmed cell death, is regulated by the sequential activation of caspases. This activation is followed by the formation of apoptotic bodies, which are then gradually engulfed either by neighboring cells or by specialized phagocytes, usually without an immune response. In contrast, necrosis is nonprogrammed cell death, which is related to the rapid loss of cell membrane integrity and inflammation. However, follow-up studies have found that certain forms of necrosis, which are referred to as necroptosis, can be programmed [14, 15]. The complex formed by receptor-interacting serine/threonine-protein kinase 3 (RIPK3) and a closely related kinase called RIPK1 (also known as the necrosome) has been identified to be the key initiator of necroptosis [16]. Recently, Wang et al. have reported that RIPK3 mediates the necroptosis of VSMCs and plays a pertinent role in AAA. Inhibition of RIPK3 could reduce the size of AAA induced by CaCl_2_ [17]. However, whether IL-37 can affect VSMC necroptosis is yet to be elucidated. Therefore, in this study, we investigated the expression of IL-37 in patients' AAA and its role in VSMC necroptosis.

## 2. Materials and Methods

### 2.1. AAA Patients

The AAA tissue samples were obtained from patients during surgical treatment for aneurysms. Normal renal aortic tissues were obtained from donors with no other vascular diseases and for kidney transplantation. The patients were treated at the Union Hospital, Tongji Medical College, Huazhong University of Science and Technology. The clinical characteristics of these patients are listed in Table 1.

We recruited 54 patients who underwent color Doppler ultrasound or helical computed tomographic angiography (CTA) between December 2018 and May 2021 at the Union Hospital Tongji Medical College. Relevant clinical examination revealed that the patients had abdominal aorta exceeding 50% of their normal vessel diameter or >3 cm, which could readily be diagnosed as AAA.

Patients with clinical cardiovascular diseases, thromboembolism, collagen disease, disseminated intravascular coagulation, advanced liver disease, renal failure, malignant disease, or septicemia or those who had undergone recent major surgery or other inflammatory diseases, as well as those who were on steroid therapy, were excluded from this study. The clinical characteristics of these patients are listed in Table 2.

This study was approved by the Ethics Committee of the Union Hospital, Tongji Medical College. Written informed consent was obtained from each patient before their enrollment in this study.

### 2.2. Human Tissue Sample Preparation, Immunohistochemistry, and Immunofluorescence

The tissue sections were routinely stained with hematoxylin and eosin or anti-human IL-37 antibodies. To determine the specific distribution of IL-37 in AAAs, we used anti-CD68, anti-CD31, and anti-*α*-SMA antibodies to identify the macrophages and VSMCs in the AAA samples, respectively. 4′,6-Diamidino-2-phenylindole (DAPI) was used as a nuclear stain. All operations were conducted in accordance with the Declaration of Helsinki. The tissue images were obtained by confocal microscopy.

### 2.3. Laboratory-Related Measurements

Fasting blood samples were collected from patients in the morning after admission. Each sample was collected in a sodium heparin vacuum container (Becton–Dickinson). These blood samples were centrifuged at 2500 × *g* for 10 min, and the plasma was stored at −80°C until further use. Moreover, mononuclear cells were separated from blood cells with the lymphocyte separation solution, collected via centrifugation with the addition of radioimmunoprecipitation assay (RIPA) buffer with 1% protease inhibitor phosphatase inhibitors and PMSF, followed by storage at −80°C until further analysis by Western blotting.

The concentrations of IL-37, IL-6, and TNF-*α* in the plasma were determined by enzyme-linked immunosorbent assay (ELISA). The minimum concentration of IL-37 was 10 pg/mL. The ELISA intra-assay and interassay coefficients of variation were 5% and 10%, respectively. The minimal detectable concentrations were 5 pg/mL for IL-6 and 8 pg/mL for TNF-*α*. The ELISA intra-assay and interassay CVs were 8% for IL-6 and 10% for TNF-*α*, respectively. All samples were measured in duplicate.

Other laboratory examination results were sourced from the Central Laboratory of Union Hospital, and the color Doppler ultrasound or CTA results were sourced from the patient's admission examination.

### 2.4. Real-Time PCR

Total RNA extracted from the cells and tissues was isolated with the TRIzol reagent (Vazyme Biotechnology, Nanjing, China) and reverse transcribed into cDNA using a reverse transcription kit as per the manufacturer's instructions. Real-time PCR was performed on the ABI 7900 System (Applied Biosystems, Foster City, CA, USA), while the SYBR Green Master Mix (Vazyme Biotechnology, Nanjing, China) was used to quantify the mRNA levels of the target genes. Each reaction was performed in triplicate, and the 2-^△△CT^ method was applied to calculate the change in each target gene. The primers used in this study were as follows:

*RIPK3*: F: GAAGGACTGAAGGAGCTAATGCA and R: TTTTGGTAGGCATTCCTGGAA, *IL-1β*: F: CCACAGACCTTCCAGGAGAATG and R: GTGCAGTTCAGTGATCGTACAGG, *GAPDH*: F: TGTTGCCATCAATGACCCCTT and R: CTCCACGACGTACTCAGCG, and *NLRP3*: F: TGAAGAGGAGTGGATGGGTTTAC and R: CTGTCTTCAATGCACTGGAATCT.

### 2.5. In Vitro Experiments

Human aortic vascular smooth muscle cells (HASMCs) were purchased from MeisenCTCC (http://www.ctcc.online), and the culture procedures were approved by the relevant ethics committee. HASMCs were cultured in DMEM supplemented with 10% fetal bovine serum in a humidified atmosphere at 37°C under a 5% CO_2_ atmosphere. As previously described, HASMCs were passaged 3–4 times at 70–80% confluence, and the cell settings were as follows: three sets of experiments, treated with PBS, AngII (50 *μ*M), AngII (50 *μ*M)+GSK'872 (5 *μ*M), AngII (50 *μ*M)+SIS3 (1 *μ*M), or AngII (50 *μ*M)+rIL37 (100 ng/mL) for 72 h. The cells (1 × 10^5^ cells/well) were seeded in a 6-well plate and transfected with the Opti-mem medium mixed with Lipofectamine 3000 as well as siRNA (50 nM) for 6 h and then replaced with the normal medium. Next, the cells were cultured for 24 h, after which AngII was added to stimulate the cells for 72 h. The cells were collected for CCK8 detection and for apoptosis-related detection.

### 2.6. Protein Extraction and Western Blotting

The cells and tissues were separated into cytoplasmic and nuclear components as per the instructions of the NE-PER Nuclear and Cytoplasmic Extraction Reagents Kit (ThermoFisher Scientific). The nuclear and cytoplasmic components were used for Western blotting, as described previously. Primary antibodies and secondary antibodies were purchased from Abcam (USA) and Cell Signaling Technology (USA).

### 2.7. Statistical Analyses

All data are presented as the mean ± SD. Data were tested for normality before processing. We used Student's *t*-test, analysis of variance, Chi-square test, and the Mann–Whitney *U* statistical test for the statistical analyses. Spearman correlation analysis was also applied to calculate the correlation between the plasma IL-37 concentration and other indicators, including age, AAA size, sex, hypertension, diabetes, smoking, systolic blood pressure, diastolic blood pressure, blood lipid levels, lipoprotein components, and fasting blood glucose levels. Simple linear regression analysis of the candidate variables such as acid, calcium, phosphorus, IL-37, IL-6, and tumor necrosis factor-*α* was also performed. In all tests, *p* < 0.05 was considered to indicate statistical significance.

## 3. Results

### 3.1. IL-37 Expression in Human AAA

**Figure 1:**
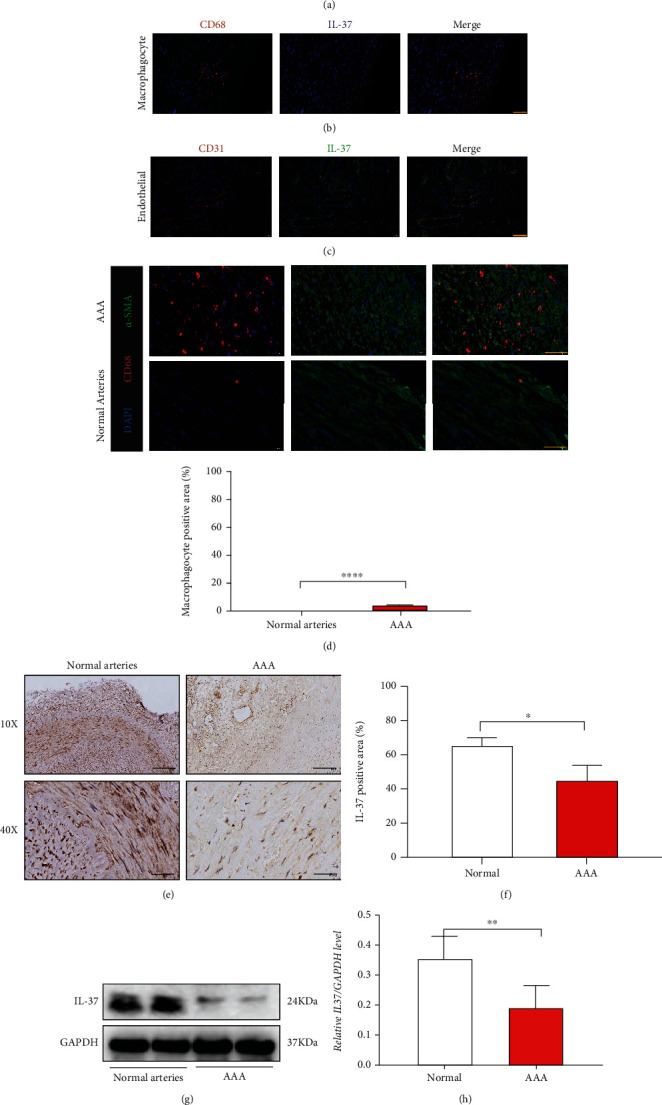
The suppression of IL-37 in human AAA. (a–c) In AAA, IL-37 was distributed in endothelial cells, macrophages, and vascular smooth muscle cells (VSMCs) and mainly concentrated in VSMCs (yellow bar = 50 *μ*m). (d) Macrophages infiltrate in AAA and fluorescence density analysis (yellow bar = 50 *μ*m). (e) Decreased IL-37 content in AAA (10x black bar = 200 *μ*m; 40x black bar = 50 *μ*m). (f) Histochemical analysis. (g) Immunoblotting observation of IL-37 expression in the vascular tissues. (h) Immunoblotting analysis. Values are presented as the means ± SD. All experiments were repeated thrice, each time with *N* = 4. ^∗^*p* < 0.05, ^∗∗^*p* < 0.01, ^∗∗∗^*p* < 0.001, and ^∗∗∗∗^*p* < 0.0001.

The basic characteristics of the patients are presented in supplemental Table 1. Anti-*α*-SMA, anti-CD68, anti-CD31, and anti-IL-37 antibodies were used for staining the AAA tissues, which revealed that although IL-37 was distributed in the VSMCs, macrophages, and endothelial cells, it was mostly concentrated in the VSMCs (Figures 1(a)–1(c)). Immunofluorescence analysis revealed a significant increase in macrophage infiltration in the AAA tissues when compared with the normal arterial tissues (Figure 1(d)). Furthermore, immunohistochemical staining and Western blot analysis showed reduced IL-37 expression in AAA than in the normal arteries (Figures 1(e)–1(h)).

### 3.2. Plasma IL-37, IL-6, and TNF-*α* in the AAA and Normal Groups

**Figure 2:**
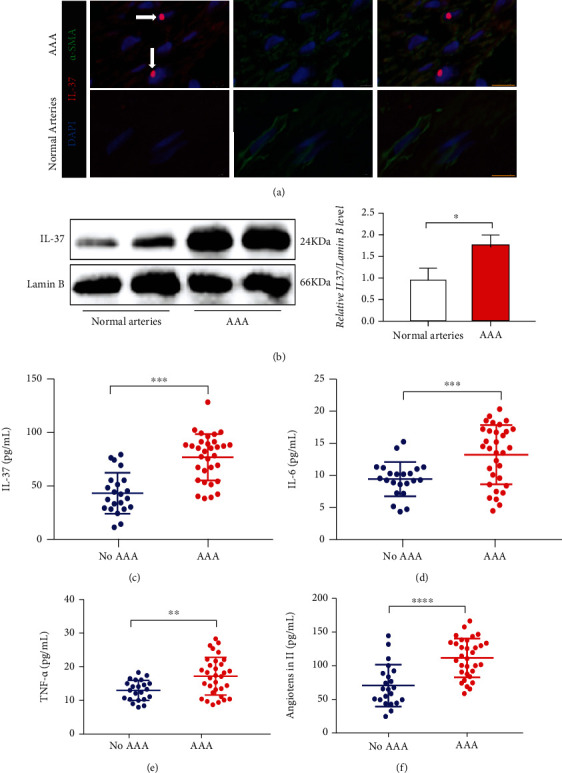
Clinical indicators of patients with non-AAA and patients with AAA. (a) In AAA samples, IL-37 translocates to the nucleus (as indicated by a white arrow, yellow bar = 10 *μ*m). (b) Expression of IL-37 in the nucleus. (c) IL-37 content in the plasma. (d) IL-6 content in the plasma. (e) TNF-*α* content in the plasma. (f) AngII content in the plasma. The basic characteristics of the patient are summarized in Table 2. ^∗^*p* < 0.05, ^∗∗^*p* < 0.01, ^∗∗∗^*p* < 0.001, and ^∗∗∗∗^*p* < 0.0001.

**Figure 3:**
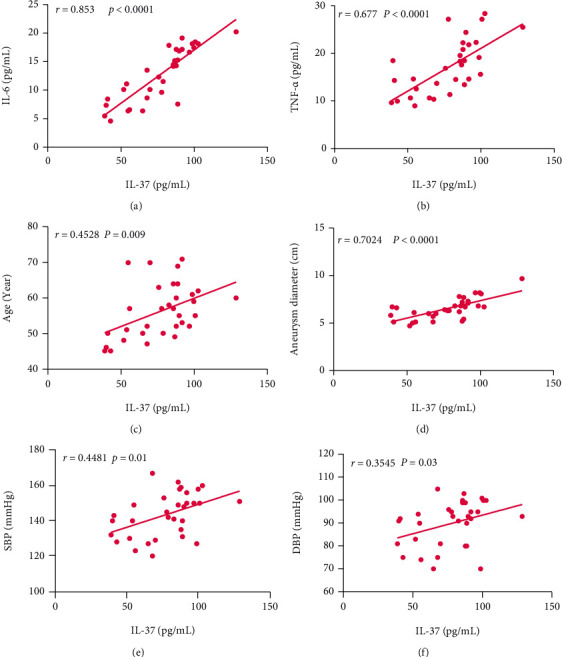
Correlation among IL-6, TNF-*α*, age, aneurysm diameter, SBP, DBP, and IL-37 (*N* = 32). (a) The plasma levels of IL-37 were positively correlated with those of IL-6 (*r* = 0.853, *p* < 0.0001). (b) The plasma levels of IL-37 were positively correlated with those of TNF-*α* (*r* = 0.677, *p* < 0.0001). (c) The plasma levels of IL-37 were positively correlated with age (*r* = 0.4528, *p* < 0.009). (d) The plasma levels of IL-37 were positively correlated with the aneurysm diameter (*r* = 0.7024, *p* < 0.0001). (e) The plasma levels of IL-37 were positively correlated with SBP (*r* = 0.4481, *p* = 0.01). (f) The plasma levels of IL-37 were positively correlated with DBP (*r* = 0.3545, *p* = 0.03).

Staining with anti-*α*-SMA and anti-IL-37 antibodies exposed that IL-37 was translocated to the nucleus in the SMCs in patients with AAA (Figure 2(a)). Similarly, nuclear protein analysis indicated increased intranuclear IL-37 protein levels in patients with AAA compared with the normal group (Figure 2(b)). The plasma levels of IL-37, IL-6, TNF-*α*, and AngII were significantly higher in patients with AAA than in the normal group. Furthermore, the levels of IL-37, IL-6, and TNF-*α* were elevated in the plasma of patients with AAA (Table 2 and Figures 2(c)–2(f)). Whether the plasma IL-37 level was related to age, aneurysm vascular diameter, and biochemical indicators, including lipid and lipoprotein components (triglycerides, high-density lipoprotein cholesterol, low-density lipoprotein, and cholesterol), fasting blood sugar, glycosylated hemoglobin, and blood pressure were subsequently assessed. Correlation analysis alluded that IL-37 was positively correlated with IL-6 TNF-*α*, age, SBP, DBP, and the inner diameter of aneurysm vessels (Table 3 and Figure 3).

### 3.3. Inhibition of RIPK3 Reduces the Apoptosis of HASMCs Induced by AngII

**Figure 4:**
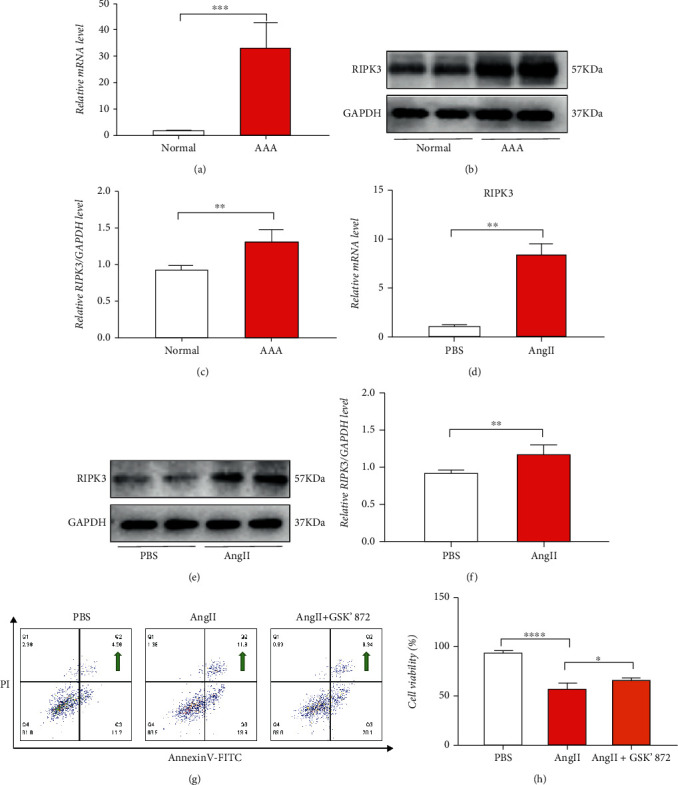
Inhibition of RIPK3 could reduce the HASMC damage. (a) The expression of RIPK3 genes in the AAA was detected at mRNA by RT-PCR (*n* = 6). (b, i) Immunoblotting observation of the RIPK3 expression in the vascular tissues. (c) Immunoblotting analysis. (d) The expression of *RIPK3* genes in the HASMCs (*n* = 5) was detected at mRNA by RT-PCR. (e, f) Immunoblotting observation of the RIPK3 expression in HASMCs. (g) Flow cytometry to detect the necroptosis (as indicated by a green arrow). (h) CCK8 was used to detect the cell viability. Values are presented as the means ± SD. All experiments were repeated thrice, each time with *N* = 4. ^∗^*p* < 0.05, ^∗∗^*p* < 0.01, ^∗∗∗^*p* < 0.001, and ^∗∗∗∗^*p* < 0.0001.

A previous study indicated that RIPK3 expression was increased in AAA [18]. This study too demonstrated that the expression of RIPK3 was increased dramatically in AAA tissues (Figures 4(a)–4(c)). *In vitro* experiments suggested that RIPK3 was also increased in HASMCs stimulated by AngII (Figures 4(d)–4(f)). Based on AngII stimulation in HASMCs, GSK'872, a RIPK3 inhibitor, was used. Flow cytometry with annexin-FITC/PI double staining was employed to measure apoptosis, and annexin-FITC+/PI+ cells represented late-stage apoptotic or necrotic apoptotic cells. Moreover, cell counting kit-8 (CCK8) was used for assessing cell viability, which revealed that the proportion of necroptotic cells was increased significantly after stimulation with AngII (Figures 4(g) and 4(h)).

### 3.4. IL-37 Regulates Inflammatory Vesicles as well as Necroptosis

**Figure 5:**
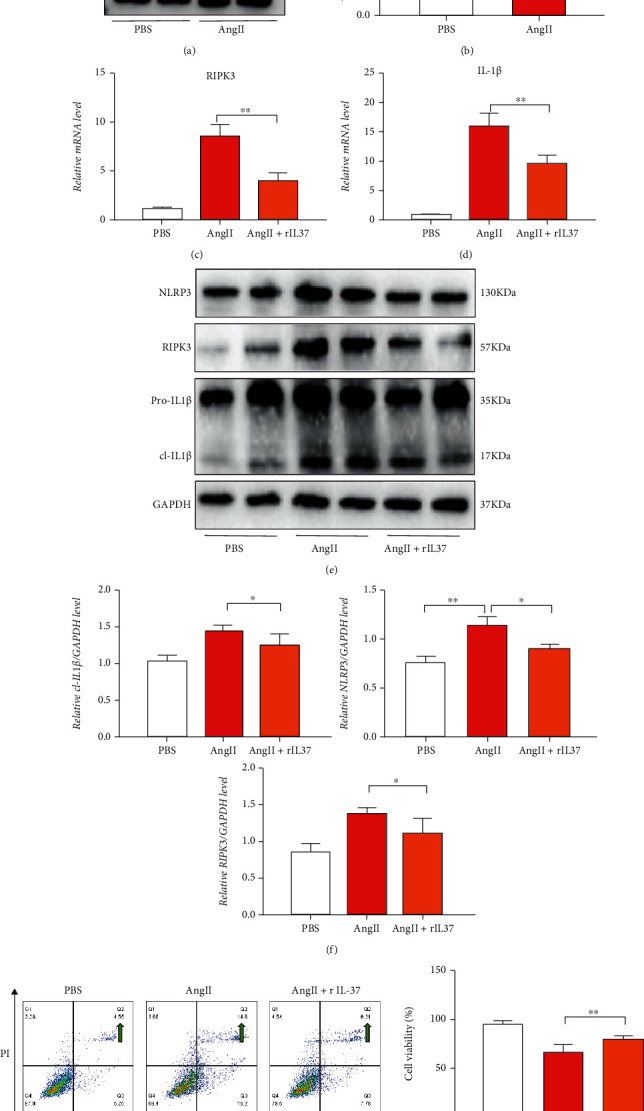
rIL-37 reduces the damage of HASMC by AngII. (a, b) Immunoblotting observation of the IL-37 expression in HASMCs. (c, d) The expression of *RIPK3 IL-1β* genes in the HASMCs (*n* = 5) was detected at mRNA by RT-PCR. (e) Immunoblotting observation of RIPK3, NLRP3, and IL-1*β* in HASMCs. (f) Immunoblotting analysis. (g) Flow cytometry to detect necroptosis (as indicated by a green arrow). (h) CCK8 was used to detect cell viability. Values are presented as means ± SD. All experiments were repeated thrice, each time with *N* = 4, ^∗^*p* < 0.05, ^∗∗^*p* < 0.01, ^∗∗∗^*p* < 0.001, and ^∗∗∗∗^*p* < 0.0001.

Immunoblotting showed that the expression of IL-37 was downregulated in HASMCs stimulated by AngII (Figures 5(a) and 5(b)). Furthermore, IL-37 appeared to be transported into the nucleus in HASMCs and was highly expressed in the nucleus by AngII stimulation (Supplemental S1 and S2). Based on AngII stimulation, RT-PCR showed that the expressions of RIPK3 and pro-IL-1*β* were decreased after rIL-37 treatment (Figures 5(c) and 5(d)). Moreover, immunoblotting suggested that the expressions of RIPK3, NLRP3, and mature cl-IL-1*β* were reduced (Figures 5(e)–5(h) and Supplemental S3). Flow cytometry and CCK8 staining demonstrated that the proportion of necroptosis (annexin-FITC+/PI+) was significantly lowered in HASMCs with rIL-37 (Figures 5(h) and 5(i)). IL-37/Smad3 complex expression was found to be increased in response to AngII stimulation (Supplemental S3). Subsequently, IL-37 or Smad3 was inhibited, and a significant increase in the mRNA and protein expressions of RIPK3, NLRP3, and IL-1*β* was observed. Flow cytometry and CCK8 assays proved that IL-37 inhibition could lead to increased necroptosis (Supplemental S4–S7).

### 3.5. IL-37 Affects the Activation of RIPK3 In Vitro

**Figure 6:**
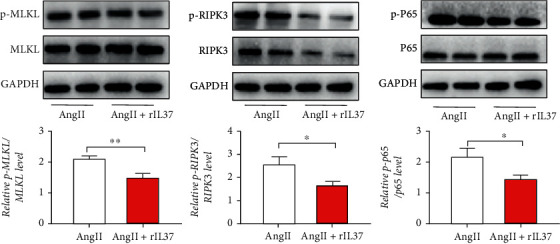
IL-37 could inhibit the activation of RIPK3 in HASMCs by AngII, followed by Western blotting to observe the expression of p-MLKL, p-RIPK3, p-p65, MLKL, RIPK3, and P65. Calculation of the ratio revealed that the phosphorylation level of MLKL, RIPK3, and P65 decreased under IL-37 (100 ng/mL) treatment. The results represent the means from at least 3 independent experiments. All values are presented as the means ± SD. All experiments were repeated thrice, each time with *N* = 4. ^∗^*p* < 0.05, ^∗∗^*p* < 0.01, ^∗∗∗^*p* < 0.001, and ^∗∗∗∗^*p* < 0.0001.

The complex formed by RIRK3 and its substrate mixed-lineage kinase domain-like (MLKL) is an important factor for the induction of necroptosis, and their phosphorylation levels are important for the formation of the necrosome complex [19]. The phosphorylated protein levels of RIPK3, MLKL, and P65 were assessed by immunoblotting HASMCs treated with rIL-37. The results showed that rIL-37 treatment downregulated RIPK3 and total P65 protein as well as their phosphorylated forms. In contrast, the levels of p-RIPK3 and p-p65 were significantly enhanced after IL-37 silencing. Interestingly, the total protein expression of MLKL remained unchanged, but the levels of the phosphorylated forms declined. This result indicated that exogenous IL-37 could inhibit p-RIPK3 and p-MLKL to alleviate the formation of necrosomes and necroptosis (Figure 6). On the contrary, after IL-37 silencing, a significant upregulation of p-RIPK3 and p-P65 by AngII was observed. Nevertheless, the total MLKL and p-MLKL levels were not significantly altered (Supplemental S7). In summary, IL-37 could either directly or indirectly regulate RIPK3 phosphorylation levels and, therefore, inhibit necroptosis.

### 3.6. IL-37 Promotes the Transition of SMCs to a Contractile Phenotype and Inhibits the Proliferation

**Figure 7:**
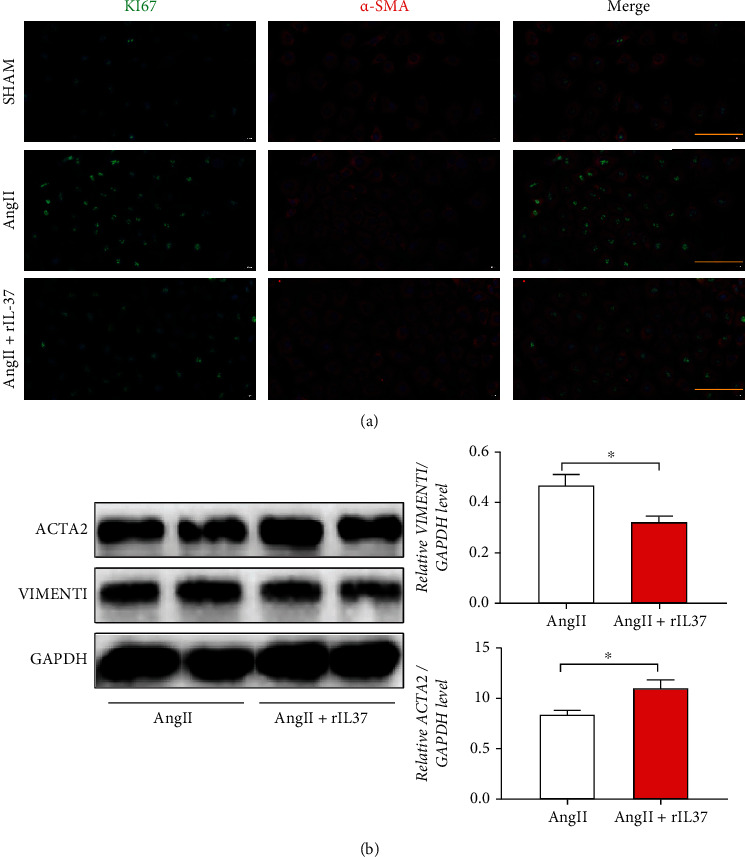
IL-37 promotes smooth muscle cell conversion to the contractile phenotype while inhibiting the proliferative phenotype. (a) Immunofluorescence detection of cell proliferation (yellow bar = 100 *μ*m). (b) Immunoblotting for SMC phenotypic transformation. ^∗^*p* < 0.05, ^∗∗^*p* < 0.01, ^∗∗∗^*p*  < 0.001, ^∗∗∗∗^*p* < 0.0001.

To confirm whether IL-37 can influence SMC proliferation, the expression of KI67 in the nucleus of SMCs was examined. Compared with the control group, enhanced proliferation of SMCs stimulated by AngII was observed, and treatment with rIL-37 inhibited SMC proliferation (Figure 7(a)). In AAA, the phenotypic transition of SMCs from contractile to proliferative as a precondition for their proliferation was a key feature [20]. To evaluate whether IL-37 could affect SMC phenotypic transformation, contractile SMC marker (ACTA2) and proliferative secretory SMC marker (Vimentin) were examined. Augmented protein expressions of systolic markers and decreased protein expressions of proliferative secretory markers were observed after rIL-37 treatment compared with no rIL-37 treatment (Figure 7(b)). These results demonstrated that IL-37 converts SMCs to a contractile phenotype and inhibits the proliferation of secretory phenotypes.

## 4. Discussion

This study demonstrated that IL-37 was substantially downregulated in the tissues of patients with AAA and was upregulated in the plasma and translocated into the nucleus in SMCs. Additionally, IL-37 modulated RIPK3, MLKL, and P65 phosphorylation, thus impacting AngII-induced and RIPK3-dependent necroptosis in SMCs. Furthermore, IL-37, which inhibited SMC proliferation, promoted the SMC phenotypic switch from a proliferative secretory to a contractile type. These findings suggest that IL-37 could be effectively exploited for the treatment of AAA.

A previous study showed that in vessels with atherosclerosis and arterial calcification, IL-37 was predominantly expressed in SMCs and that its expression was significantly higher in the arteries than in the other tissues [8]. Our results indicated that in AAA tissues, IL-37 expression was most abundant in SMCs but was also expressed in endothelial cells and macrophages although at lower levels. Similar to the atherosclerotic vessels, macrophage infiltration was seen in AAA vessels, but there was a trend toward decreasing abundance of IL-37 in AAA samples. The disease processes underpinning AAA have long been considered a restricted manifestation of advanced atherosclerosis [21]. It has been published that systemic vascular branches are abnormal in patients with AAA. In fact, proteolysis, inflammation, and SMC apoptosis of the vascular branch are the key factors that cause AAA [22]. Furthermore, AAA pathogenesis is influenced by genetic, epigenetic, and external stimuli [23]. Therefore, the diametrically opposed abundance of IL-37 expression in the arteries representative of these two diseases alludes that the pathophysiological mechanisms involved in atherosclerosis and AAA development may be quite different.

In this study, the level of plasma IL-37 was measured in patients with AAA and healthy controls and it was found to be increased in AAA. In abnormally activated cells, such as those stimulated by AngII or LPS, IL-37 is released by the cells into the peripheral blood and suppresses the activation of immune cells. It has been reported that IL-6 and TNF-*α* can be secreted by leukocytes and SMCs and that the plasma concentrations gradually increase as the size of the abdominal aorta increases [24]. Correlation analysis showed that IL-37 was positively correlated with IL-6, TNF-*α*, age, aneurysm diameter, SBP, and DBP. The main effect of IL-37 was to reduce excessive inflammatory reaction via negative feedback, which played an important role in the innate and adaptive immune mechanisms. IL-37 present in the peripheral environment of patients with AAA is speculated to also act as negative feedback to regulate inflammation and delay aneurysm progression. Our findings showed that IL-37 in SMCs is translocated into the nucleus, and that the nuclear expression is increased in AAA. Interestingly, total IL-37 was downregulated in the AAA tissues. Endogenous IL-37 not only can translocate into the nucleus but also be released outside the cell to participate in the immune response. Owing to the decrease of total IL-37 in SMCs and the increase of intracellular inflammation, IL-37 in the nucleus cannot completely suppress inflammation, which aggravates AAA. This is the first study to have described the expression of IL-37 in patients with AAA.

AngII can trigger RIPK3–MLKL-mediated necroptosis by activating the FAS/FASL signaling pathway in renal tubular cells [25]. Consistent with the findings in the AAA tissues, RIPK3 expression in SMCs was upregulated upon AngII stimulation *in vitro*. The proportion of cells undergoing necroptosis was reduced after RIPK3 was inhibited using GSK'872. These findings imply that AngII-mediated SMC necroptosis is RIPK3-dependent. RIPK3 promoted AAA by inducing SMC necrosis and inflammation. A study on CaCl2-induced AAA showed that the aneurysm diameter was significantly reduced in RIPK3-knockout mice, as was the smooth muscle cell necroptosis rate [17]. Our results are consistent with previously published data and assert that in AngII-induced smooth muscle injury model, RIPK3 may serve as a therapeutic target to alleviate SMC injury in different AAA models.

Our data show that IL-37 regulates necroptosis and inflammation in SMCs. As in the case of AAA tissues, downregulation of IL-37 expression stimulated by AngII was observed in HASMC. Quite unexpectedly, RIPK3, NLRP3, and IL-1*β* were significantly suppressed in SMC by AngII after rIL*-*37 treatment. Pro-IL-1*β* can be sheared into cl-IL-1*β* by the NLRP3 inflammasome complex [26]. Exogenous IL-37 binds to IL-18Ra and recruits IL-1R8. Formation of the IL-37/IL18RA/IL1R8 complex inhibits the inflammatory pathways (NF-*κ*B/p65, MAPK, NLRP3, etc.) and activates the anti-inflammatory pathways (AMPK, FOX0, STAT3, etc.) [27]. Hence, it is hypothesized that IL-37 inhibits NLRP3 expression and other transcriptional pathways that increase IL-1*β* expression by forming coreceptors. For the first time, IL-37/Smad3 complex binding was shown to be increased in SMCs following AngII stimulation. Furthermore, inhibition of endogenous IL-37 or Smad3 increased necroptosis as well as inflammation in our study. Smad3, when phosphorylated, inhibits cytotoxicity by binding to DNA and inhibiting dendritic cell (DC) and macrophage activation. It has been reported that Smad3 protects vascular integrity and suppresses vascular inflammation [28]. As the only cytokine that can bind to Smad3, IL-37 can phosphorylate as well as dephosphorylate Smad3, both of which exert an inhibitory effect on inflammation. Similarly, necroptosis of SMCs was significantly enhanced after inhibiting IL-37 or Smad3. These results indicate that IL-37 could alleviate necroptosis of SMCs via Smad3.

Feng et al. reported that the administration of rIL-37 could inhibit the expression and activation of MLKL in the liver cells by lowering INF-*γ*/TNF-*α*, thereby alleviating liver damage and fibrosis [29]. This finding suggests that IL-37 could also affect MLKL function and expression. In our study, p-RIPK3 and p-MLKL were reduced in AngII-induced SMCs by rIL-37 treatment. As a kinase, activation of RIPK3 promotes the phosphorylation of MLKL to form a RIPK3–MLKL complex (also known as a necrosome) to induce necroptosis. Activated MLKL is translocated into the cell membrane, thus forcing the cell membrane to rupture [30]. As IL-37 did not affect total MLKL protein, it is hypothesized that it is likely to regulate RIPK3 and, thus, influence the phosphorylation of MLKL. The NF-*κ*B family comprises five proteins: RelA (p65), RelB, c-Rel, NF-*κ*B1 (p50), and NF-*κ*B2 (p52), which are activated and translocated into the nucleus to regulate the expression of inflammatory factors. In DCs and macrophages stimulated by LPS, IL-37 reduces p65 phosphorylation levels. In SMCs, IL-37 acts like the one described above. However, further experiments are needed to elucidate how IL-37 affects the phosphorylation of these proteins in SMC.

In AAA, the smooth muscle cells are transformed from a contractile to a proliferative secretory type. The proliferation is dominated by proliferative secretory [31], and AngII-induced AAA also exhibits a similar performance [32]. As an experimental method of inducing AAA, AngII can enhance the above transformation of VSMCs via AT1R. In *in vitro* experiments, high-dose AngII (>10^−7^) promoted SMC proliferation. At a concentration of 10^−5^ (50 *μ*M), the level of SMC proliferation was significantly enhanced. Under the stimulation of AngII, the SMC phenotype switch may be regulated by RIPK3, NLPR3, inflammatory factors, etc. Interestingly, this proliferation was reversed after treatment with rIL-37. In one study, overexpression of IL-37 reduced SMA and Vimentin expression in the aorta in atherosclerosis. Similarly, exogenous IL-37 treatment reduced AngII-induced SMC proliferation, upregulated the expression of ACTA2 (contractile), and downregulated the expression of Vimentin (proliferative secretory). Succinctly, IL-37 inhibits SMC inflammation, necroptosis, and proliferation, thus converting SMC to a contractile phenotype, and also inhibits the proliferative secretory phenotype, thereby inhibiting AAA formation.

Nevertheless, this study has certain limitations. First, we could not collect normal abdominal aortic vessels because of ethical issues; thus, renal arteries from healthy donors were used as controls. Second, the sample size is small; more patients should be recruited to determine the relationship between plasma concentration of IL-37 and AAA recovery. Third, in *in vitro* cell experiments, AngII stimulation may not entirely simulate the damage in human AAA; hence, other AAA models should be applied.

## Figures and Tables

**Table 1:** Clinical characteristics of patients.

Characteristics	Normal arteries groups	AAA groups	*p* value
Age (y)	57 ± 7.6	62 ± 4.7	NS
Sex (male/female)	6/0	6/0	NS
Hypertension, n (%)	1 (16.7)	5 (83.3)	^∗∗^
Diabetes, n (%)	1 (16.7)	1 (16.7)	NS
Smoking, n (%)	0	6 (100)	^∗∗∗∗^
SBP (mmHg)	125 ± 13.7	148 ± 10.2	^∗∗^
DBP (mmHg)	76 ± 3.9	91 ± 7.6	^∗∗^
GLU (mmol/L)	5.57 ± 1.40	5.75 ± 1.38	NS
TC (mmol/L)	3.70 ± 0.25	3.65 ± 0.17	NS
TG (mmol/L)	1.43 ± 0.74	1.53 ± 0.58	NS
LDL-C (mmol/L)	2.13 ± 0.18	2.20 ± 0.15	NS
HDL-C (mmol/L)	1.09 ± 0.15	1.20 ± 0.20	NS

The data are given as the mean ± SD, percentages, or numbers. SBP: systolic blood pressure; DBP: diastolic blood pressure; TC: total cholesterol; TG: triglyceride; LDL-C: low-density lipoprotein cholesterol; HDL-C: high-density lipoprotein cholesterol. ^∗^*p* < 0.05, ^∗∗^*p* < 0.01, ^∗∗∗^*p* < 0.001, and ^∗∗∗∗^*p* < 0.0001.

**Table 2:** Clinical characteristics of the patients with AAA.

Characteristics	No AAA	AAA	p value
Age (y)	50 ± 8.1	56 ± 7.6	NS
Sex (male/female)	14/8	28/4	^∗^
Hypertension, n (%)	4 (18.2)	22 (68.8)	^∗∗∗^
Diabetes, n (%)	6 (27.3)	8 (25)	NS
Smoking, n (%)	3 (13.6)	18 (56.3)	^∗∗^
SBP (mmHg)	125 ± 14.7	143 ± 12.6	^∗∗∗∗^
DBP (mmHg)	76 ± 11.8	90 ± 9.9	^∗∗∗∗^
GLU (mmol/L)	5.95 ± 1.2	5.83 ± 1.2	NS
TC (mmol/L)	4.32 ± 0.6	4.40 ± 0.8	NS
TG (mmol/L)	1.12 ± 0.5	1.20 ± 0.7	NS
LDL-C (mmol/L)	2.21 ± 0.7	2.19 ± 0.6	NS
HDL-C (mmol/L)	1.29 ± 0.3	1.32 ± 0.4	NS
Aneurysm diameter (cm)	None	6.6 ± 1.1	^∗∗∗∗^

The data are presented as the mean ± SD or the number of patients. SBP: systolic blood pressure; DBP: diastolic blood pressure; GLU: glucose; TC: total cholesterol; TG: triglyceride; LDL-C: low-density lipoprotein cholesterol; HDL-C: high-density lipoprotein cholesterol; ^∗^*p* < 0.05, ^∗∗^*p* < 0.01, ^∗∗∗^*p* < 0.001, and ^∗∗∗∗^*p* < 0.0001.

**Table 3:** Spearman's correlation analysis.

	TNF-*α*	IL-6	IL-37
Age (y)	0.271	0.263	0.453^∗∗^
Aneurysm			
Diameter (cm)	0.506^∗∗^	0.649^∗∗∗∗^	0.702^∗∗∗∗^
SBP (mmHg)	0.439^∗^	0.593^∗∗∗^	0.448^∗^
DBP (mmHg)	0.423^∗^	0.535^∗∗^	0.355^∗^
GLU (mmol/L)	0.218	0.174	0.189
TG (mmol/L)	0.148	0.187	-0.090
TC (mmol/L)	-0.084	-0.073	-0.092
LDL-C (mmol/L)	0.055	0.076	-0.053

^∗^ *p* < 0.05, ^∗∗^*p* < 0.01, ^∗∗∗^*p* < 0.001, and ^∗∗∗∗^*p* < 0.0001.

## Data Availability

Data are available on request.

## Supplementary Materials

Supplementary Materials S1: translocation of IL-37 in HASMCS to the nucleus in response to AngII stimulation (yellow bar = 50 *μ*m). S2: immunoblotting, total IL-37 expression in the nucleus. S3: COIP,IL-37/Smad3 complex was increased in AngII group. S4: flow cytometry, SIS3 promoted necroptosis in HASMCs. S5: RT-PCR, increased expression of NLPR3, RIPK3, and IL-1*β* after knockdown of IL-37. ^∗^*p* < 0.05, ^∗∗^*p* < 0.01, ^∗∗∗^*p* < 0.001, and ^∗∗∗∗^*p* < 0.0001. S6: immunoblotting, increased expression of NLPR3, RIPK3, and IL-1*β* after knockdown of IL-37. S7: increased rates of apoptosis and damage after knockdown of IL-37. ^∗^*p* < 0.05, ^∗∗^*p* < 0.01, ^∗∗∗^*p* < 0.001, and ^∗∗∗∗^*p* < 0.0001. S8: immunoblotting, upregulation of p-RIPK3 and p-p65 phosphorylation levels after knockdown of IL-37. ^∗^*p* < 0.05, ^∗∗^*p* < 0.01, ^∗∗∗^*p* < 0.001, and ^∗∗∗∗^*p* < 0.0001.
