# Supplementary material for: IL-37 Expression in Patients with Abdominal Aortic Aneurysm and Its Role in the Necroptosis of Vascular Smooth Muscle Cells
Source: Oxid Med Cell Longev. 2022 May 11;2022:1806513. doi: 10.1155/2022/1806513 (PMC9117036; doi:10.1155/2022/1806513)
Supplement: Supplementary Materials — S1: translocation of IL-37 in HASMCS to the nucleus in response to AngII stimulation (yellow bar = 50 μm). S2: immunoblotting, total IL-37 expression in the nucleus. S3: COIP,IL-37/Smad3 complex was increased in AngII group. S4: flow cytometry, SIS3 promoted necroptosis in HASMCs. S5: RT-PCR, increased expression of NLPR3, RIPK3, and IL-1β after knockdown of IL-37. ∗p < 0.05, ∗∗p < 0.01, ∗∗∗p < 0.001, and ∗∗∗∗p < 0.0001. S6: immunoblotting, increased expression of NLPR3, RIPK3, and IL-1β after knockdown of IL-37. S7: increased rates of apoptosis and damage after knockdown of IL-37. ∗p < 0.05, ∗∗p < 0.01, ∗∗∗p < 0.001, and ∗∗∗∗p < 0.0001. S8: immunoblotting, upregulation of p-RIPK3 and p-p65 phosphorylation levels after knockdown of IL-37. ∗p < 0.05, ∗∗p < 0.01, ∗∗∗p < 0.001, and ∗∗∗∗p < 0.0001. [file 1806513.f1.docx]

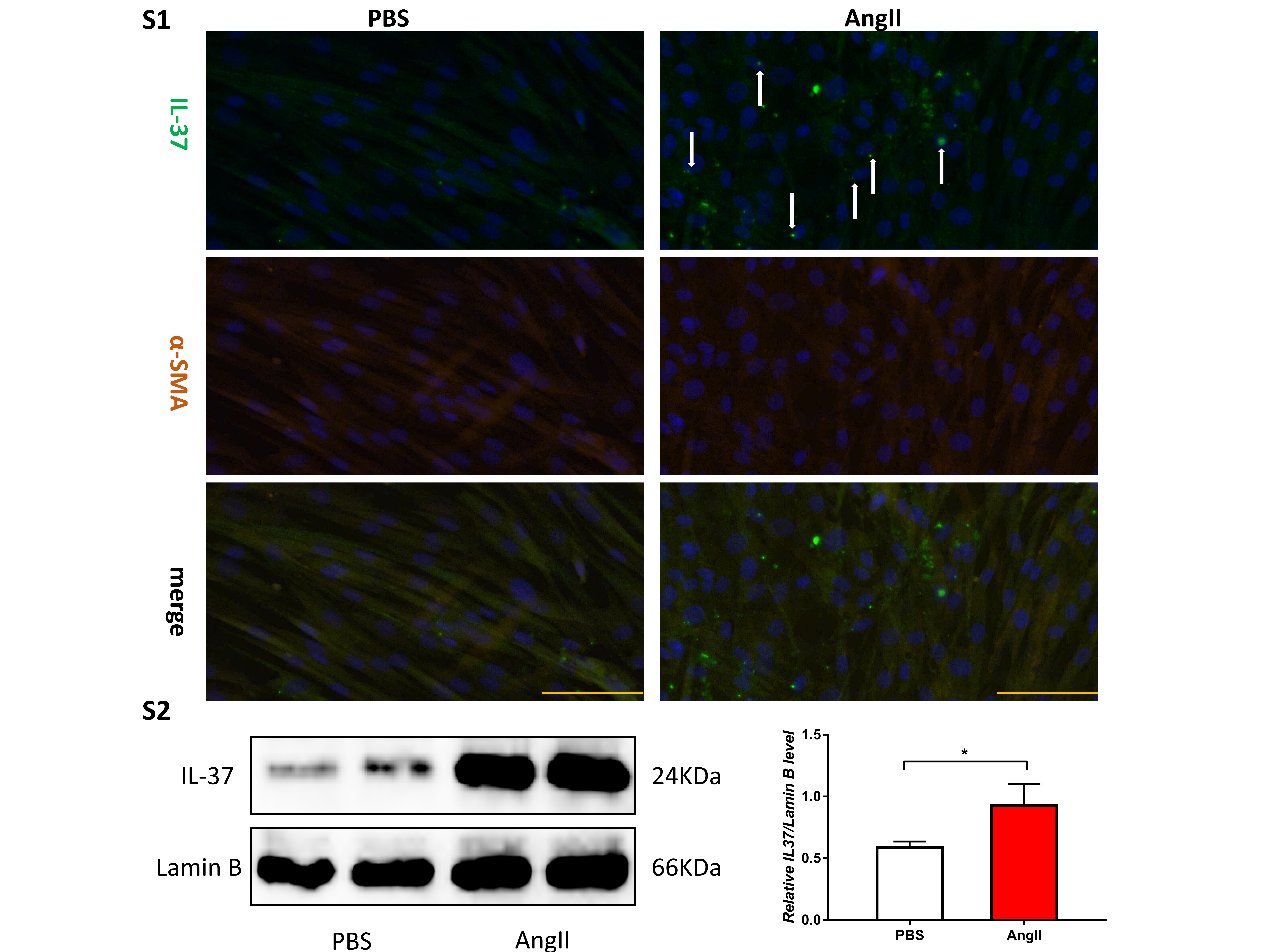


S1: Translocation of IL-37 in HASMCS to the nucleus in response to AngII stimulation (yellow bar=50μm)

S2: Immunoblotting, total IL-37 expression in the nucleus


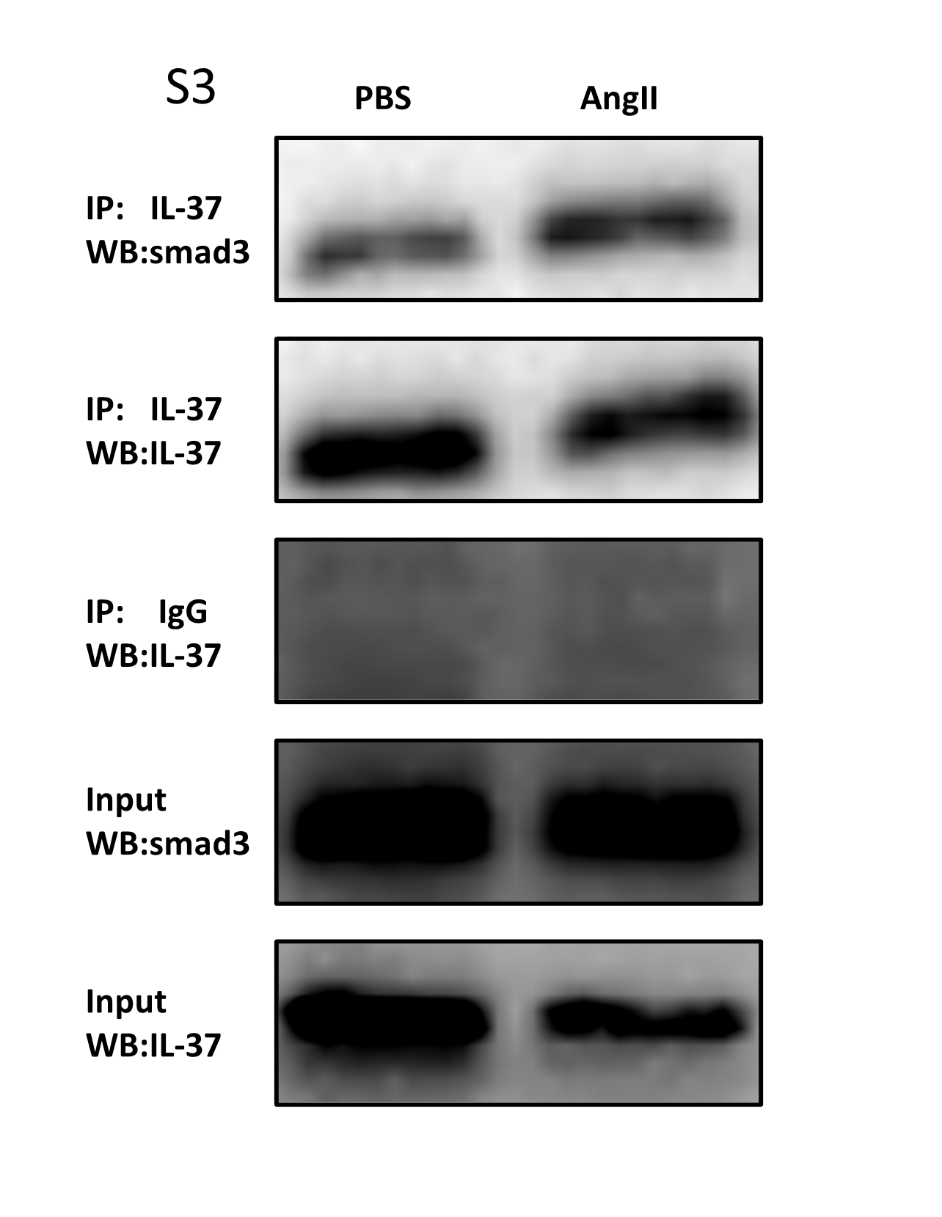


S3: COIP,IL-37/smad3 complex was increased in AngII group


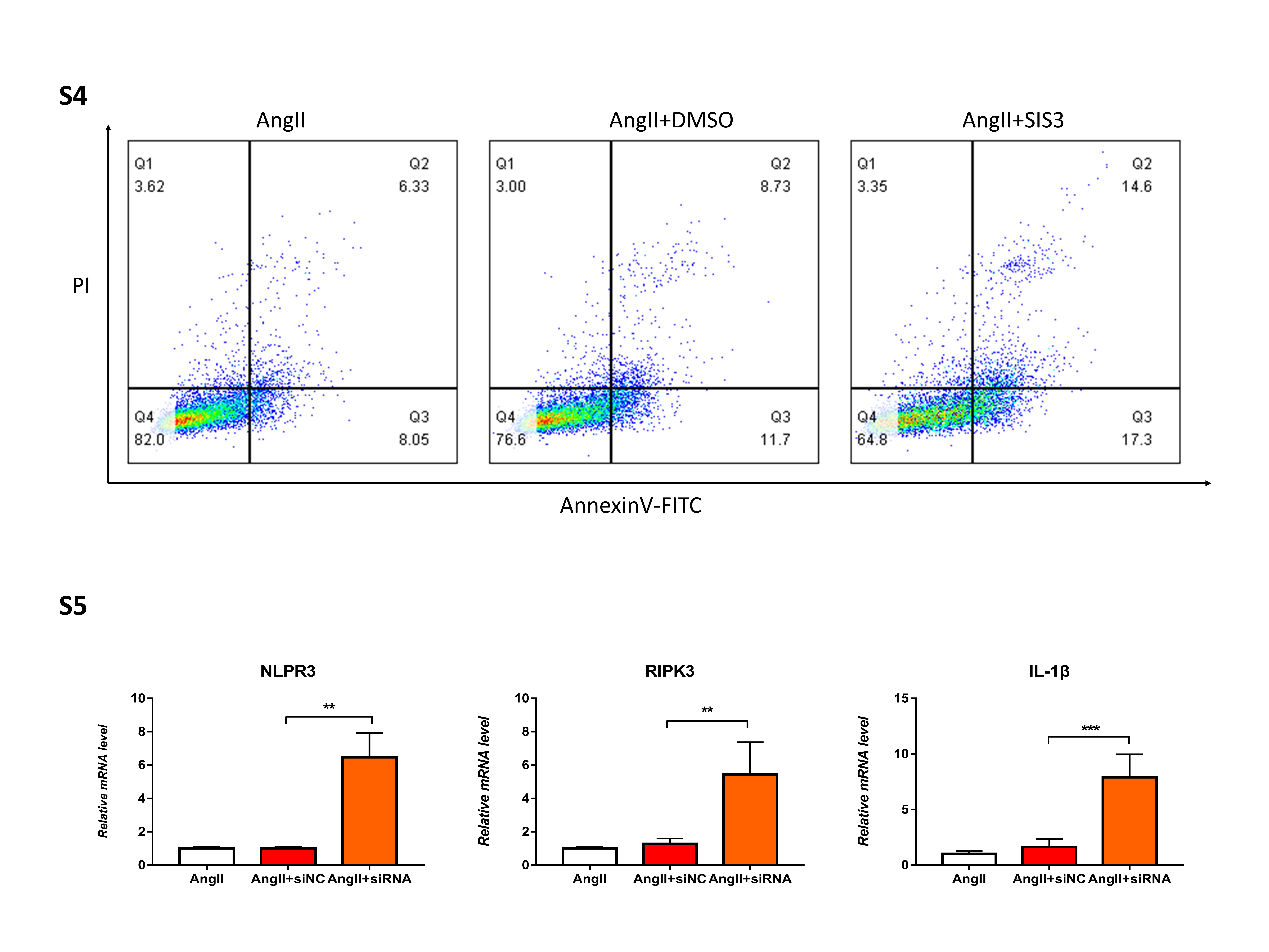


S4: Flow cytometry, SIS3 promoted necroptosis in HASMCs

S5: RT-PCR, increased expression of NLPR3, RIPK3, IL-1β after knockdown of IL-37

*p<0.05 **p<0.01 ***p<0.001 ****p<0.0001


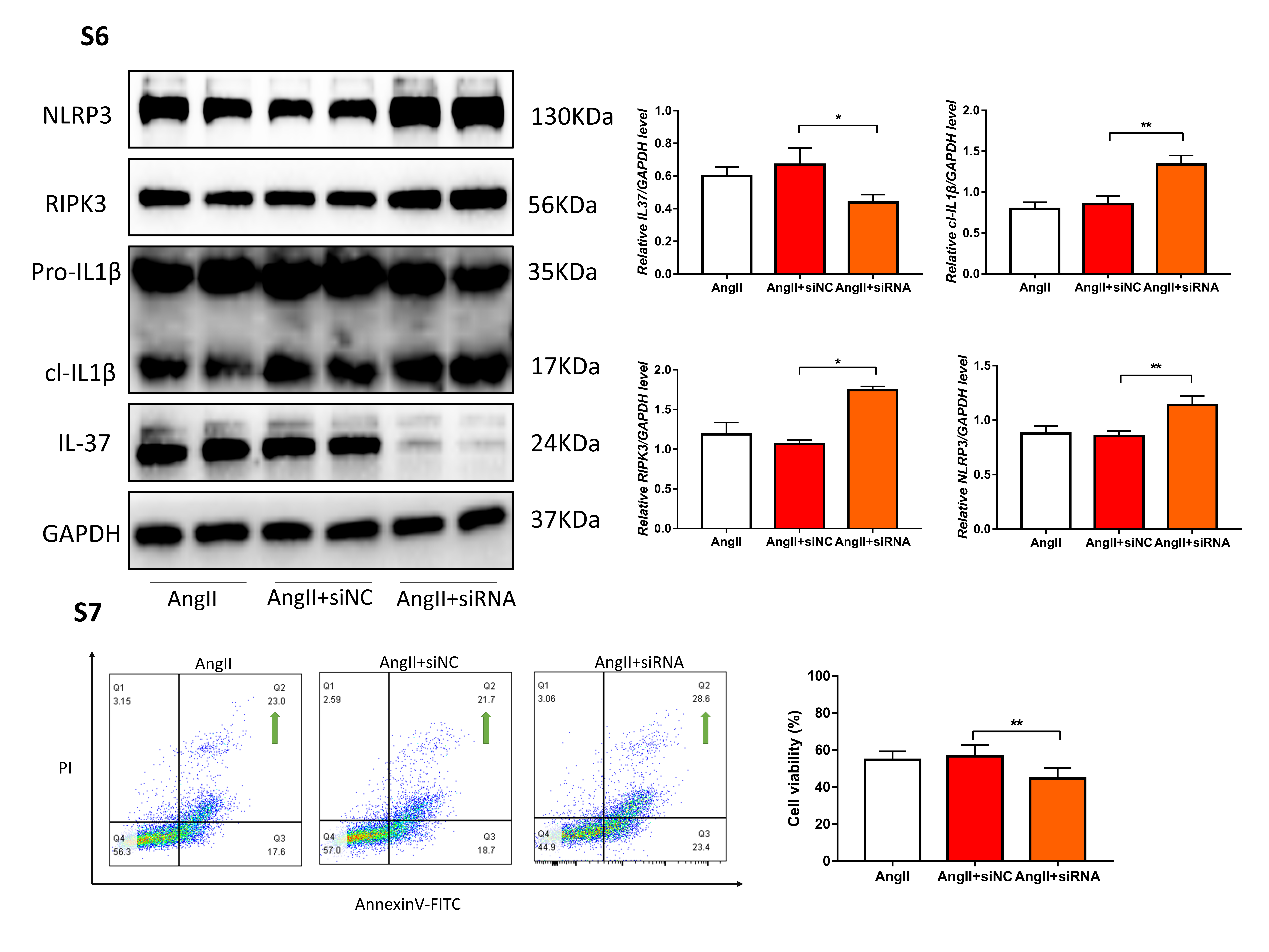


S6: Immunoblotting, increased expression of NLPR3, RIPK3,IL-1β after knockdown of IL-37

S7: Increased rates of apoptosis and damage after knockdown of IL-37

*p<0.05 **p<0.01 ***p<0.001 ****p<0.0001


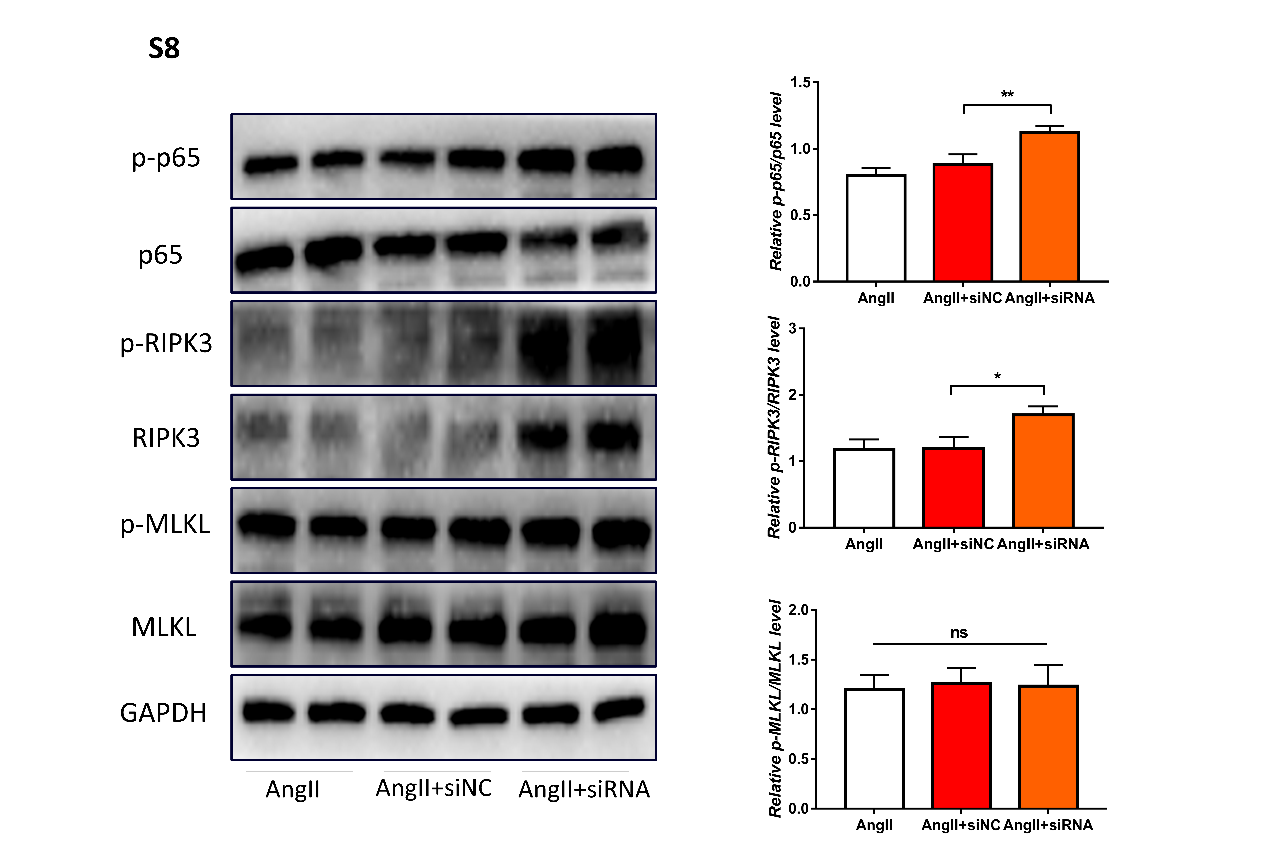


S8: Immunoblotting, upregulation of p-RIPK3 and p-p65 phosphorylation levels after knockdown of IL-37

*p<0.05 **p<0.01 ***p<0.001 ****p<0.0001
